# Clinician’s Guide to Epitranscriptomics: An Example of N^1^-Methyladenosine (m^1^A) RNA Modification and Cancer

**DOI:** 10.3390/life14101230

**Published:** 2024-09-25

**Authors:** Ana Kvolik Pavić, Josipa Čonkaš, Ivan Mumlek, Vedran Zubčić, Petar Ozretić

**Affiliations:** 1 Department of Maxillofacial and Oral Surgery, University Hospital Osijek, Josipa Huttlera 4, 31000 Osijek, Croatia; kvolik-pavic.ana@kbco.hr (A.K.P.); zubcic.vedran@kbco.hr (V.Z.); 2 Faculty of Medicine Osijek, Josip Juraj Strossmayer University of Osijek, Josipa Huttlera 4, 31000 Osijek, Croatia; imumlek@gmail.com; 3 Laboratory for Hereditary Cancer, Division of Molecular Medicine, Ruđer Bošković Institute, Bijenička Cesta 54, 10000 Zagreb, Croatia; jconkas@irb.hr

**Keywords:** epitranscriptomics, RNA modifications, m1A, reader, writer, eraser, cancer

## Abstract

Epitranscriptomics is the study of modifications of RNA molecules by small molecular residues, such as the methyl (-CH_3_) group. These modifications are inheritable and reversible. A specific group of enzymes called “writers” introduces the change to the RNA; “erasers” delete it, while “readers” stimulate a downstream effect. Epitranscriptomic changes are present in every type of organism from single-celled ones to plants and animals and are a key to normal development as well as pathologic processes. Oncology is a fast-paced field, where a better understanding of tumor biology and (epi)genetics is necessary to provide new therapeutic targets and better clinical outcomes. Recently, changes to the epitranscriptome have been shown to be drivers of tumorigenesis, biomarkers, and means of predicting outcomes, as well as potential therapeutic targets. In this review, we aimed to give a concise overview of epitranscriptomics in the context of neoplastic disease with a focus on N^1^-methyladenosine (m^1^A) modification, in layman’s terms, to bring closer this omics to clinicians and their future clinical practice.

## 1. Introduction to the Indirect Flow of Genetic Information

The term “The Central Dogma”, coined by Frances Crick and published in 1958, states that “DNA makes RNA, and RNA makes protein” [1]. Although the wording has changed through the decades, the idea was that the transfer of information in molecular biology is linear. However, we now know that the molecular biology of life is not as straightforward (and simple) as that [2]. DNA can transfer its information to DNA, or RNA, and RNA can transfer the information back to DNA by a process of reverse transcription. There are also many layers of information transfer that influence this process and can terminate, silence, or enhance the transcription. One of the ways this can be achieved in a cell is by adding small biochemical tags to the nucleic bases. When the tag is present, changes to polarity and hydrophobicity brought by the tag change the secondary and tertiary structures of nucleic acids. These changes modify the way enzymes react with their nucleic acid substrates and produce a downstream effect. Epigenetics is a study of these reversible changes to the molecule of DNA that do not change the nucleotide composition of DNA [3]. Similarly, epitranscriptomics studies the reversible tags on RNA molecules. There are over 200 different ways an RNA molecule can be modified. Some of them are frequent, and some are rare and species-specific. Nevertheless, they are all important for the normal functioning of a cell, as well in pathologic processes such as malignant transformation.

## 2. Epitranscriptomics, a New Layer of Genetic Information Post-Transcriptionally Encoded into the RNA

Epitranscriptomics, which studies post-transcriptional changes in RNA, is growing fast. With hundreds of studies focusing on the mapping and functions of specific or global RNA modifications, it is difficult for a clinician to fully understand this topic. Epitranscriptomics could roughly be divided into two processes: RNA editing and RNA modification. RNA editing is a post-transcriptional change in the nucleotide sequence of an RNA molecule. The best examples are cytidine into uridine (C-to-U) and adenosine into inosine (A-to-I) base changes, which are considered as RNA editing by deamination. RNA editing events occurring in the coding region of a gene (CDS) can lead to changes in amino acid composition of a protein or in a premature STOP codon [4]. RNA modification is a process where certain residues within RNA molecules, most commonly the C or N atom of a nitrogenous base, are modified by small molecular residues such as the methyl (-CH_3_) group [5]. This review aims to give, in layman’s terms, an overview of research methods and recent findings in the field of RNA modification, with a primary focus on N^1^-methyladenosine (m^1^A) modification of various RNA molecules and its significance in cancer research and practice.

### 2.1. The Origin of a New Omics

The development of DNA and RNA sequencing techniques in the 1960s and 1970s led to a race to translate and categorize the human genome. Scientists quickly realized that DNA is much more than a set of repeating nucleic bases that give a recipe for making a protein. A base can be modified by methylation, and histone, a protein around which the DNA wraps to give the chromosome a more compact shape, can be modified as well. These changes could be functionally relevant, stable, and heritable [3].

**Figure 1:**
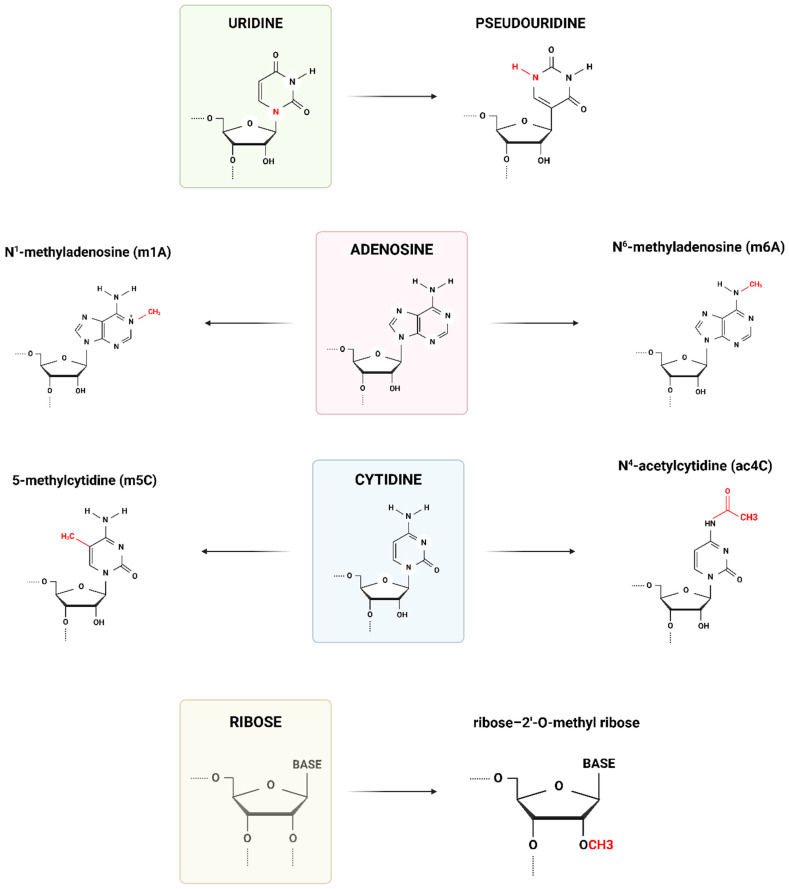
Comparative molecular structures of unmodified RNA nucleosides and ribose with some of the most common RNA modifications. Created with https://BioRender.com (accessed on 16 May 2024).

The first modified RNA base was pseudouridine (Ψ) discovered in 1957 [6]. It is distinct from uridine (U) because it has a C-C bond, rather than the usual C-N glycosyl bond. This allows Ψ to be more conformationally flexible than U and changes the way RNA molecules stack together [7]. Other modified bases were discovered, but these modifications were first considered static [8]. Studies on evolutionally conserved non-coding RNAs identified overall ratios of methylation [9]. As time progressed, numerous RNA modifications were identified, together with enzymes that introduce them into the RNA molecules. The study protocols varied and were plagued by false positives or negatives or contamination by other types of RNA [10,11]. Currently, there are over 200 known natural and artificially produced modified RNA bases [12]. For example, the most common natural epitranscriptomic mark is N^6^-methyladenosine (m^6^A). It is evolutionally highly conserved and appears in various cell types from *Archaea* to humans. Although only 0.1–0.2% of adenosines in mammalian cells are modified in the m^6^A manner, m^6^A still accounts for more than half of all the modified adenosines [13]. Some of the other more common modifications are the aforementioned Ψ, which is present almost as much as m^6^A, 5-methylcytidine (m^5^C), N^1^-methyladenosine (m^1^A), N^4^-acetylcytidine (ac^4^C), and ribose 2′-O-methylations (Nm) [11] (Figure 1).

**Figure 2:**
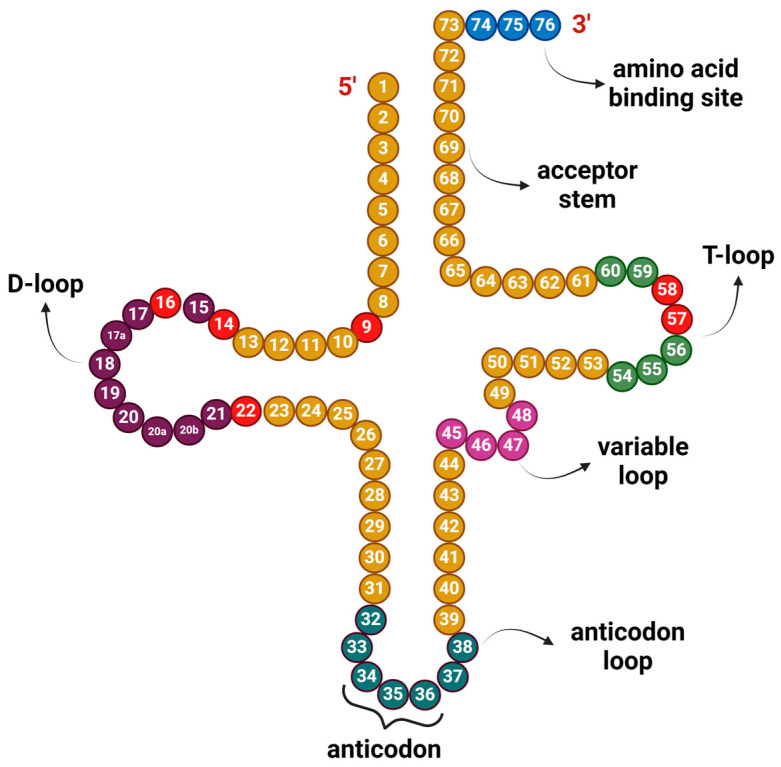
Location of m^1^A modifications within the cloverleaf structure of tRNA molecule (in red). D-loop (in purple) is named after dihydrouridine (D or DHU), a modified nucleotide generally present in this region. The anticodon loop contains the anticodon (in dark green), which recognizes and binds to a specific codon on mRNA during protein translation. Variable loop (in magenta) varies from 3–21 bases and is used for classification of tRNAs. T-loop or TΨC loop (in light green) contains modified uridine, a pseudouridine (Ψ). Acceptor stem is formed by the base pairing of the 5′ end and the 3′ end of tRNA. Amino acid binding site or CCA tail (in blue) is a cytosine–cytosine–adenine motif at the 3′ end of tRNA, and amino acid is covalently bonded to it by aminoacyl tRNA synthetase. Created with https://BioRender.com (accessed on 28 June 2024).

These appear less frequently throughout the epitranscriptome, sometimes only in specific positions, such as m^1^A which appears in bases 9, 14, 16, 22, 57, and 58 of the transfer RNA (tRNA) (Figure 2) [14]. Comparatively, m^6^A is ten times more common than m^1^A [15].

tRNA is the most modified RNA molecule in the cell [16]. The tRNA modifications allow for the stability of the classical clover-like structure and are present in both the anticodon loop as well as other structural regions. The first two letters of the codon bind the second and third positions of the anticodon following the Watson–Crick base pairing, but the last letter does not always follow suit. This is called wobble pairing [17]. The tRNA modification in this spot is an important regulator that allows the anticodon to pair with non-canonical codons and expand the tRNA vocabulary. The m^1^A brings a positive charge to the RNA molecule and thus affects its structure and defines its relationship with proteins and codon–anticodon pairings. It can be found in six locations on tRNA, with positions 9 and 58 being essential for the clover-like structure [18]. It is also present in rRNA where it affects the formation of the 60S unit of the ribosome [19] and in mRNA where it affects transcript stability [20].

### 2.2. Readers, Writers, and Erasers

Although the scientific community has known about modified RNA bases for decades, the exact mechanism by which they were made was not fully understood. Methyltransferase complexes (“writers”), a class of enzymes that catalyze the transfer of the -CH_3_ group from the methyl donor S-adenosyl-L-methionine (SAM) to their substrates, target specific motifs in the RNA molecule and introduce the mark in the conserved locations. Specific enzymes (“readers”) bind the modified bases and produce downstream effects by recruiting signaling pathways that can enhance translation, cause decay of the mRNA, mediate alternative splicing, or facilitate the inclusion of alternative exons. These marks are not permanent but can be removed via “eraser” demethylase complexes which remove -CH_3_ groups from nucleic acids. The functions of some of these enzymes overlap, with several enzymes affecting the same spot or targeting multiple RNA modifications [21].

**Figure 3:**
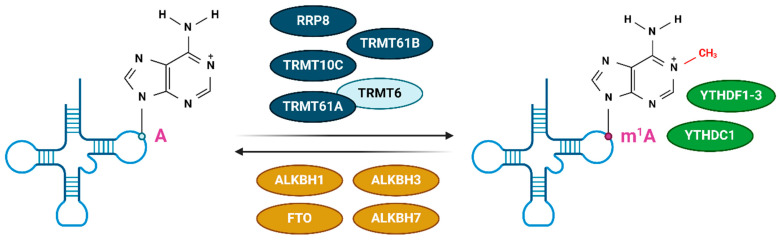
Some of the known readers (green), writers (blue), and erasers (ochre) of the m^1^A modification of the tRNA molecule. Created with https://BioRender.com (accessed on 17 May 2024).

The major writer complex of the m^1^A modification is the tRNA (adenine-N^1^-)–methyltransferase complex built of two subunits, TRMT61A and TRMT6 [14,22]. TRMT61A contains SAM which functions as a methyl group donor. TRMT6 does not have a methyl donor group but is crucial for tRNA binding. This complex is located in the cytosol and recognizes the tRNA T-loop and mRNA with a T-loop-like GUUCRA nucleotide motif [15]. Other known m^1^A writers are TRMT61B which methylates mitochondrial 16S rRNA, TRMT10C which can also write an m^1^G mark, and ribosomal RNA-processing protein 8 (RRP8) which is found in the nucleus and methylates 28S rRNA (Figure 3) [23].

The first discovered demethylase was alpha-ketoglutarate-dependent dioxygenase FTO (also known as fat mass and obesity-associated protein) that was initially described as an m^6^A demethylase but was later found to demethylate m^1^A as well [21]. The m^1^A is primarily removed by a family of AlkB homolog (ALKBH) demethylases, among which ALKBH1 demethylates the majority of cytoplasmatic tRNA. ALKBH3 can demethylate both m^1^A and m^3^C in tRNA. ALKBH7 functions in mitochondria where it can demethylate m^1^A and m^2^G [24].

So far, the dedicated m^1^A reader has not been discovered. There are several YT521-B homology (YTH) domain-containing proteins (YTHDF1, YTHDF2, YTHDF3, and YTHDC1) that primarily bind the m^6^A but have been found to bind also to m^1^A sites but with a weaker affinity [25]. Their function has not been properly explained [26]. Also, it is difficult to delineate the effects of a reader enzyme that binds two modifications, one of which (m^6^A) is 10 times more common than the other one [27].

### 2.3. m^1^A—From Physiology to Oncology

Under physiological circumstances, the methyl group in m^1^A has a positive electrostatic charge, which can obstruct the Watson–Crick base pairing with uridine [28], and therefore m^1^A modifications can affect RNA processing, secondary and tertiary structure formation and stability, and interactions within a single RNA or between different RNAs as well as their partner proteins, which all have an impact on the physiological roles of RNAs [23].

For example, m^1^A58 methylation in tRNA_i_^Met^ is essential for maintaining the stability of its inverted L-shape tertiary structure [29], while m^1^A9 methylation of mitochondrial tRNA^Lys^ is indispensable for its proper folding into the standard cloverleaf structure [30].

The regulatory effects of m^1^A in translation vary depending on the type of modified RNA and can impact both the translation initiation and elongation processes. To improve translation initiation, as mentioned previously, m^1^A58 stabilizes the initiator methionine tRNA_i_^Met^, while the level of m^1^A58 in mitochondrial tRNA^Lys^ strongly increases protein synthesis [31]. Similarly, m^1^A-modified tRNAs are more preferentially recruited by active polyribosomes to promote translation [32]. Increased translation initiation and efficiency are also linked to the m^1^A modifications found in the 5′ untranslated region (5′UTR) of mRNA [33]. To destabilize the 5′UTR secondary structures and to promote the initiation step of protein translation, m^1^A can interfere with intramolecular RNA base pairing [34]. On the other hand, ribosomal scanning or the binding of a releasing factor to modulate translation are the mechanisms by which m^1^A modifications in a gene’s protein-coding sequence (CDS) induce an inhibitory effect on translation [34].

While the presence of m^1^A is involved in the structural thermostability of tRNAs, the deficiency of m^1^A writers induces thermosensitivity of tRNAs. For example, *ALKBH1* knockdown fails to rescue tRNA from cleavage [35], while *ALKHB3* overexpression induces the formation of tRNA fragments (tRFs) [36].

m^1^A modifications and their regulators are involved in many different cellular processes, such as proliferation, invasiveness, cell metabolism, senescence, and cell death, which are all associated with tumor formation and progression [27].

The proliferation of cancer cells has been found to be promoted by m^1^A regulators, such as TRMT6, TRMT61A, and ALKBH3, in colorectal cancer [37], gastric cancer [38], glioma [39], hepatocellular cancer (HCC) [40], and prostate cancer [41].

In breast and ovarian cancers, ALKBH3 induces the abundance of m^1^A-modified colony-stimulating factor 1 (*CSF1*) mRNA, which enhances its translation initiation and cancer cell invasiveness [42], while it can also promote cancer cell invasion through destabilization of tRNAs [36].

The oncogenesis of HCC is promoted by the elevated levels of m^1^A58-modified tRNAs by the TRMT6/TRMT61A complex, which increases peroxisome proliferator-activated receptor delta (PPARδ) translation and stimulates cholesterol synthesis, resulting in the activation of the Hedgehog pathway and thus initiated self-renewal of HCC stem cells [43]. Through modifying the expression of m^1^A-methylated ATP synthase F1 subunit delta (*ATP5D*) mRNA, ALKBH3 stimulates the glycolysis of cancer cells [44].

In lung and bladder cancers, *ALKBH3* knockdown results in the induction of senescence and cell cycle arrest, by increasing the expression of cell-cycle arrest proteins p27 and p21 and modulating NADPH oxidase and TWEAK/Fn14 signaling with induced vascular endothelial growth factor A (*VEGFA*) expression [45,46]. *ALKBH3* knockdown also induces cell cycle arrest or apoptosis depending on the tumor protein p53 (*TP53*) gene status in non-small-cell lung carcinoma cells, in which *TP53* knockout shifts from cell cycle arrest to apoptosis induction [47].

Hodgkin lymphoma cells were found to have *ALKBH3* hypermethylation of the promoter CpG island and its transcriptional silencing, which has also been linked to poor clinical outcomes in Hodgkin lymphoma patients [48].

## 3. Detection of Epitanscriptomic Marks (When You Want to Get Your Hands Dirty)

It has been decades since the discovery of the first modified RNA base [6], but epitranscriptomics has only recently become a tool in diagnostics and disease prediction, and not just a basic science research topic. At first, each mark needed to be studied separately. The data were quantitative, meaning only a small amount of modification in a certain molecule could be obtained. Newer methods allowed for more precise positioning of the modified nucleoside but were still limited to detecting a single modification at a time. In 2019, Khoddami et al. published the RBS-seq study protocol that can simultaneously detect multiple RNA modifications in a single specimen at a single-base resolution [49]. With a growing number of published studies, better detection tools, and more complete epitranscriptomic maps, epitranscriptomics is ready to enter clinical practice [50]. In this section, we will present relevant diagnostic tools as well as sources of samples from which one can detect and study epitranscriptomic marks in a clinical setting.

Liquid samples such as blood, urine, and saliva are readily available and noninvasive [51]. Tissue samples can be obtained during surgical procedures; however, these procedures are invasive, and due to tumor heterogeneity, a specific sample sometimes does not represent the entire tumor mass. Also, obtaining healthy tissue for the control group can be ethically problematic, while obtaining urine or saliva samples from healthy individuals is much easier. After collection, tissue samples need to be stabilized to preserve the integrity of the RNA. Traditional formalin fixation degrades the RNA, and the results obtained from fixed RNA samples may be unreliable [52]. Instead, tissue samples must be fresh-frozen by immersion into liquid nitrogen or preserved in commercially available RNA-stabilizing solutions such as RNA*later*^®^ (Ambion, Austin, TX, USA), Allprotect^®^ Tissue Reagent (Qiagen, Hilden, Germany), or PAXgene^®^ Tissue STABILIZER (PreAnalytix, Hombrechtikon, Switzerland) [53].

There are many experimental methods used for studying RNA modifications [54], some of which could be more or less easily applied in clinical practice, and those will be presented next in more detail.

### 3.1. Liquid Chromatography

Liquid chromatography is a method that uses differences in net charge, polarity, or hydrophobicity to separate modified from unmodified bases in a stationary phase. Sensitivity can be enhanced by using radioactive labeling and cleavage. When coupled with mass spectrometry (liquid chromatography–mass spectrometry or LC-MS), the specimen mass can be greatly reduced. Fang et al. used the hydrophilic interaction liquid chromatography tandem mass spectrometry method to quantify ten different modified nucleosides in the urine of patients with breast cancer [55].

### 3.2. Dot Blot

Dot blot is a semi-quantitative method that uses base-specific antibodies to detect modified bases that are blotted directly on the membrane [56]. It can be used as another layer of proof. In the study by Cheray at al., the authors used five different approaches to determine the levels of m^5^C in glioblastoma multiforme to avoid contamination and biases, one of which was dot blot [57].

### 3.3. Reverse Transcription (RT)

Reverse transcription (RT) uses the enzyme reverse transcriptase to produce a complementary strand of DNA (cDNA) from an isolated mRNA. The modified bases can be detected because their presence inhibits primer extension, which allows for context-specific positioning of the modification site. It is always necessary to compare a sample RNA with an unmodified control RNA to account for structural stops [54].

### 3.4. Next-Generation Sequencing (NGS)

Next-generation sequencing (NGS), or massive parallel sequencing (MPS), is an upgrade on the reverse transcription technology. RNA sequencing or RNA-seq is a technique in which RNA is directly converted into a library of cDNAs which are afterward sequenced in a high-throughput manner. The information obtained this way is processed by bioinformatics software and compared with reference transcripts so a modification can be positioned within the genome. This process increases both speed and accuracy compared with traditional sequencing methods, e.g., Sanger sequencing [58]. Over time, many methods have been developed that focus on RNA modifications. For example, MeRIP-seq (methylated RNA immunoprecipitation sequence) is a method specifically aimed at detecting m^6^A modification in the RNA [59] and m^1^A-ID-seq is designed for transcriptome-wide m^1^A mapping [60].

However, not only fresh tissue and liquid samples can be of use in epitranscriptomic studies. Clinically the most used technique for detecting tumor biomarkers, immunohistochemistry (IHC) can also be used to stain slices of formalin-fixed, paraffin-embedded (FFPE) tissue blocks with antibodies specific to each RNA modification [61]. This gives important information about the cellular and subcellular distributions of a certain RNA modification in tissues. However, since the chemical structure of different RNA modifications is frequently extremely similar, antibodies must be thoroughly tested in a specific model system to obtain the most accurate results [62].

## 4. Bioinformatics in Epitranscriptomic Research (Or When You Don’t Want to Get Your Hands Dirty)

Next-generation sequencing produces vast amounts of raw data that are becoming more and more difficult to navigate and analyze. Bioinformatics allows us to sift through all these data and find connections that would have been impossible to find manually. In the field of epitranscriptomics, several online databases emerged to categorize RNA modifications, such as RMBase, MODOMICS, and RNAmod (Table 1). RMBase v3.0 contains transcriptome-wide landscapes of more than 500 samples from 13 animal species, plants, bacteria, and yeasts. It contains data about the precise location of over 1.3 million RNA modifications, data regarding RNA-binding proteins, and single-nucleotide variants [63]. MODOMICS is a database focusing on the structures and lifecycle of modified RNA bases as well as enzymes that modify RNA [64]. RNAmod is a web-based platform for the meta-analysis and functional annotation of modifications on mRNAs [65].

However, in addition to studying the modified nucleotide(s) by itself/themselves, it is also important to study the enzymes that regulate it, or more commonly, the genes that encode for those enzymes. And this is where the cancer genome databases come into play.

In 2006, the US National Cancer Institute’s (NCI) Center for Cancer Genomics (CCG) and the National Human Genome Research Institute (NHGRI) started a pilot project TCGA (The Cancer Genome Atlas) that sought to sequence genetic mutations responsible for cancer [66]. Initially, they focused on three types of cancer in 500 patients. With the development of high-throughput genome analysis, the project grew to include 33 cancer types, thousands of cases, whole-exome and transcriptome-wide sequencing, and whole-genome sequencing of 10% of cases. The TCGA also contains clinical information such as survival data, TNM grade, tumor stage, and demographic data. The majority of TCGA data are in the public domain, allowing scientists all over the world to search for specific genes or proteins and compare them to their own study sample or healthy controls.

The GEO (Gene Expression Omnibus) is another repository of high-throughput gene expression data [67]. This database allows easy submission and retrieval of the data via indexing and linking the engine like the one used by the PubMed database [71]. The submission can remain private for a maximum of six months or after the publication of a manuscript, after which it becomes public. The release of the experimental data to the public is one of the requirements set by the funding agencies or scientific journals.

The GTEx (Genotype-Tissue Expression) project is an open-access project managed by the National Institutes of Health (NIH) that collects and studies healthy tissue samples from deceased transplant donors [72]. The collected data include whole-genome sequencing (WGS) of each donor as well as gene expression profiles (via RNA sequencing) of each of the 54 healthy tissue sites.

Since the amount of available omics data is ever-increasing, it is necessary to learn how to sift and navigate through them easily, even for a person without any bioinformatic knowledge. Several online tools have been developed that allow user-friendly exploratory research based on existing genomic databases, primarily TCGA, of which GEPIA and UALCAN are the most used. GEPIA (Gene Expression Profiling Interactive Analysis) is an interactive tool that allows differential expression analysis, survival analysis, and similar gene detection. Easier data retrieval bridges the gap between big data and cancer researchers [48]. UALCAN (The University of ALabama at Birmingham CANcer data analysis Portal) is also an interactive interface that allows researchers to search for specific genes in the context of a specific cancer type. It can also show over- and under-expressed genes by cancer types, positively and negatively correlated genes, methylation profiles, and survival data with graphic representations and heatmaps [70].

The vast amount of data from freely accessed datasets made bioinformatics an important tool in epitranscriptome research. With knowledge of a specific reader, writer, or eraser protein and the gene that encodes it, researchers can retrieve expression profiles with available demographic and clinical data, survival analysis, levels of methylation, or search for other genes that are positively or negatively correlated with the gene of interest. This allows anyone anywhere to benefit from the already available data, set a hypothesis, and prove a concept in silico, without the hindrance and additional resources needed to collect the samples and process it on its own.

Table 2 lists some of the studies which are based on in silico analyses of public omics data for m^1^A-regulated genes in different types of cancers, of which most aim to find new (or a combination of) diagnostic and prognostic biomarkers.

## 5. Application of Epitranscriptomics in Clinical Oncology Practice (“with a Stethoscope”)

Since epitranscriptomic marks are omnipresent in cells, they are probably involved in the majority, or even all, of cellular processes. These marks are one of the ways in which environmental factors can interplay an inherited genetic code and tip the scale toward tumorigenesis. So far, epigenetic marks have tentatively been used as a biomarker, a prognostic sign, and a treatment target. Fang et al. studied levels of modified RNA nucleosides in the urine of breast cancer patients and healthy controls and developed a nomogram for the detection of early-stage breast cancer [55]. Zheng et al. studied serum levels of modified nucleosides in patients with colorectal adenomas, colorectal cancer, and healthy controls and showed differences in the levels of the majority of nucleosides and even a gradual decrease in 2′-O-methyluridine (Um) and 2′-O-methylguanosine (Gm) concentrations from healthy controls to adenomas to colorectal carcinomas, which is consistent with tumor development from the healthy mucosa of the gut to premalignant and finally malignant lesions [79].

These few examples show the potential application of epitranscriptomic research in clinical practice. However, the published papers are still just dots that need to be connected. Many laboratories develop their own methods and protocols, and experimental data are prone to contamination and bias [11]. The results need to be systematized and reproducible before they can enter mainstream healthcare [50]. The cost of introducing another layer of laboratory tests needs to be weighed against the benefit provided for the patient in terms of better disease prediction or change in treatment.

The pharmacological treatment of epitranscriptome imbalances is still in its infancy [80]. Multiple low-molecular-weight compounds have been studied as far back as 2015 [81]. DNA methyltransferase inhibitor treatment approved by the U.S. Food and Drug Administration (FDA), such as 5-azacytidine (azacitidine) [82] or 5-aza-2-deoxycytidine (decitabine) [83], could also demethylate RNA molecules, but it is unclear whether the treatment response is a result of DNA or RNA demethylation [84]. Since the m^6^A mark is most abundant in human RNA and METTL3-METTL14 upregulation is a hallmark of cancer [85], some biotech companies have already developed METTL3 inhibitors. The first prototypes of METTL3 inhibitors were SAM structural analogs [86]. However, the only cell-permeable inhibitor of METTL3/14 described to date is nucleoside analogue sinefungin, which inhibits the majority of methyltransferases [87].

Due to the poor cell permeability and binding affinity of adenosine analogs, non-nucleoside-selective METTL3 inhibitors are developed to overcome these limitations, providing better cellular uptake and stronger binding capabilities [88]. Non-nucleoside METTL3 inhibitors, such as UZH1a [89] and STM2457 [90], have been developed through structure-based drug design and high-throughput screening, showing promising results in cellular assays and animal models [86]. These inhibitors demonstrate the therapeutic potential of targeting METTL3 in diseases like cancer. An METTL3 inhibitor, UZH1a, was identified by the Caflisch group and has shown high nanomolar potency and good selectivity and cellular activity where in vitro demonstrated a reduction exclusively in the m^6^A/A ratio within acute myeloid leukemia, osteosarcoma, and kidney cancer cells. Additionally, a protein thermal shift assay revealed selectivity toward other RNA methyltransferases, indicating that levels of other RNA modifications such as m^1^A, m^6^Am, and m^7^G remained unchanged [89]. Interestingly, STORM Therapeutics announced phase 1 clinical trials of its METTL3-targeting drug STC-15 for refractory acute myeloid leukemia (AML) in 2020, after promising results in mice [91], meaning that STC-15 is the first METTL3 inhibitor to enter clinical trials and the initial results are expected to be released shortly (ClinicalTrials.gov: NCT05584111). However, STC-15 triggers innate immune pathways and suppresses tumor growth in preclinical cancer models. It also boosts the effectiveness of anti-PD-1 therapy, resulting in a sustained and powerful anti-tumor immune response [91]. However, despite the development and testing of numerous METTL3 inhibitors, no inhibitors targeting other METTL family proteins have been reported to date. Interestingly, Wang et al. screened FDA-approved drugs and identified thiram as a potent inhibitor of the TRMT6/TRMT61A methyltransferase complex, which suppressed liver cancer stem cell self-renewal and reduced oncosphere formation in HCC cell lines in vitro, as well as inhibited tumor growth in vivo [43]. The m^1^A methyltransferase complex, TRMT6/TRMT61A, is overexpressed in HCC and is associated with poor prognosis. Through their screening, Wang et al. discovered three drugs—thimerosal, phenylmercuric acetate (PMA), and thiram—that inhibit the TRMT6/TRMT61A interaction. Notably, thiram significantly reduced HCC growth in preclinical models, indicating its potential as a therapeutic agent [43]. Combining thiram with the PPARδ antagonist GSK3787 synergistically inhibited liver cancer with high m^1^A methylations, without affecting other RNA modifications like m^1^G and Ψ. Thiram also significantly inhibited glioma and reduced Lewis lung carcinoma metastasis in mice, suggesting potential use as an angiogenesis inhibitor [43]. However, high doses caused liver damage and severe toxicity in animal models [43,92]. Further preclinical studies are needed to assess thiram’s therapeutic potential for cancer treatment, particularly for HCC patients.

Treatment options for RNA demethylases have also been studied. Chen et al. studied rhein, a naturally occurring compound, that reversibly binds to FTO’s active site in vitro and increases overall m^6^A levels in cells with minimal cytotoxicity [93]. While most research has focused on examining the effect of FTO inhibitors on m^6^A levels, since FTO also demethylates m^1^A, the findings could potentially have therapeutic implications for regulating m^1^A modification in tumors. For example, the inhibition of FTO-dependent demethylation could offer a therapeutic approach to block the development of tyrosine kinase inhibitor (TKI) resistance. Exposure to 25 µM rhein significantly inhibited cell proliferation in TKI-resistant leukemia cells compared to parental controls, suggesting that combining rhein with TKI treatments might be more effective in reducing cell viability and colony formation, as well as overcoming drug resistance, than using TKI treatments alone [94]. However, rhein also interacts with the demethylases ALKBH2 and ALKBH3, which target m^1^A and m^3^C modifications, respectively, but it engages different binding sites for inhibiting ALKBH and FTO [95]. Aside from rhein, several more FTO inhibitors have been identified to date. Among these is MO-I-500, an FTO pharmacological inhibitor, which significantly inhibited breast cancer survival and colony formation in vitro [96]. Likewise, a range of FTO inhibitors have been found to exert significant anti-tumor effects in AML, primarily by suppressing cell proliferation and enhancing apoptosis. FB23-2, by mimicking FTO depletion, markedly inhibits proliferation, promotes differentiation and apoptosis in human AML cell lines, and significantly impedes AML progression in xenotransplanted mice [97]. R-2-hydroxyglutarate (R-2HG) has similar effects, which reduces *MYC/CEBPA* transcript stability and suppresses relevant pathways, with similar effects seen in glioma, suggesting R-2HG’s potential for targeting FTO/m^6^A/MYC/CEBPA signaling in FTO-high cancers [98]. Apart from cell proliferation and apoptosis effects, two potent FTO inhibitors, CS1 and CS2, reduce leukemia stem cell self-renewal and enhance T cell cytotoxicity by reprogramming immune responses, highlighting FTO’s role in cancer stem cell self-renewal and immune evasion, as well as its potential as a cancer therapy target [99]. Moreover, another FTO inhibitor, FTO-04, inhibits neurosphere formation in patient-derived glioblastoma stem cells without affecting healthy neural stem cells [100]. On the other hand, FTO inhibitor Dac51, a recently discovered FB23 analog, increases CD8 + T cell infiltration and enhances PD-L1 immunotherapy efficacy, generating significant anti-tumor effects [101]. Aside from the FTO inhibitors, ten years ago, Nakao et al. identified an ALKBH3 inhibitor HUHS015 [102]. This compound inhibits the growth of the prostate cancer cell line and reduces tumor burden in mouse xenograft models [103]. However, HUHS015 has not yet been tested in clinical trials. To sum up, ongoing research and development of high-quality probes and biomarkers, along with non-cytotoxic inhibitors, hold promise for advancing personalized treatments and improving outcomes in cancer, particularly for therapy-resistant cases with abnormal m^1^A patterns.

On the other hand, while specific inhibitors for each YTH family member are still undiscovered, structural biology research has provided essential insights for designing small-molecule YTH inhibitors, again with emphasis on the m^6^A modification. Micaelli’s group recently discovered that ebselen, an organoselenium compound identified through high-throughput screening for ligands in the m^6^A pocket, binds to YTHDF proteins without discrimination between the three YTHDF paralogs and interferes with their recognition of m^6^A-modified RNAs [104]. In a separate study, Hong et al. used structure-based virtual screening to identify tegaserod as a potential YTHDF1 inhibitor. Tegaserod blocked YTHDF1’s binding to m^6^A-modified mRNAs, reducing the viability of patient-derived AML cells and extending survival in xenograft mouse models [105]. To sum up, designing a drug that targets a specific RNA modification and produces the desired effect without side effects rippling through the epitranscriptome is a challenge because these modifications are involved in so many cellular processes. A dysregulation of m^6^A can promote both the pro-oncogenic environment as well as affect immune cells involved in anti-tumor response [106]. However, advances of highly selective epitranscriptomic drugs are sure to find a place in the future of precision medicine [107]. Table 3 summarizes key m^1^A RNA methylation inhibitors and their testing phases across various cancer types.

## 6. Conclusions

The molecular biology of life is already too complex, so adding any new layer of genetic information flow can only bring more noise and confusion. However, epitranscriptomics, one of the latest branches of science that investigates the entire complement of modifications of RNA molecules, is an emerging field of basic research with promising possibilities of clinical applications. RNA modifications such as pseudouridylation and methylation (e.g., N^1^-methyladenosine, m^1^A) influence the stability, splicing, translation, and subcellular localization of RNA molecules. Therefore, these modifications can alter gene expression patterns and aberrant RNA modifications can lead to uncontrolled cell growth, resistance to apoptosis, and increased metastatic potential. On the other hand, enzymes involved in adding, removing, or reading RNA modifications (writers, erasers, and readers) are potential therapeutic targets, and inhibitors of these enzymes could modulate RNA modification patterns and suppress tumor growth. Furthermore, there is already plenty of evidence that specific RNA modification patterns can serve as biomarkers for early cancer detection, prognosis, and treatment response monitoring. All these have proven that the dawn of epitranscriptomic medicine has already broken, and in that light, medical, radiation, and surgical oncologists should be aware of novel perspectives that could advance their practice and ensure better care for their patients.

## Figures and Tables

**Table 1:** Some of the online resources used for in silico epitranscriptomic research (accessed on 21 May 2024).

Resource	Web Address	Content	Reference
RMBase v3.0	https://rna.sysu.edu.cn/rmbase3/	An online platform with eight modules that provides resources and tools for analyzing RNA modifications. It contains data on thousands of epitranscriptomes pertaining to 73 RNA modifications in 63 species.	[63]
MODOMICS	https://genesilico.pl/modomics/	Database of RNA modifications, their structures, biosynthetic pathways, modifying enzymes, and location.	[64]
RNAmod	https://rnainformatics.org.cn/RNAmod/	Up-to-date database of naturally occurring RNA modifications that is constantly updated after the initial publication in 1994.	[65]
TCGA	https://www.cancer.gov/ccg/research/genome-sequencing/tcga	Genomic project that sequenced genomes of 33 cancer types and matched healthy tissue samples from over 20,000 individuals.	[66]
GEO	https://www.ncbi.nlm.nih.gov/geo/	Public functional genomics array- and sequence-based data repository.	[67]
GTEx	https://gtexportal.org/home/	Public access database of whole genomes and transcriptomes of 54 healthy tissues collected from organ donors.	[68]
GEPIA	http://gepia.cancer-pku.cn/	A web server that provides user-friendly pan-cancer and cancer-specific analyses of expression and clinical data of 9736 tumors and 8587 normal tissue samples from the TCGA and the GTEx projects.	[69]
UALCAN	https://ualcan.path.uab.edu/	Web portal that provides user-friendly analysis of mRNA and protein expression, methylation, and survival data as well as visualization of the TCGA datasets.	[70]

GEO—Gene Expression Omnibus; GEPIA—Gene Expression Profiling Interactive Analysis; GTEx—Genotype—Tissue Expression; RMBase—RNA Modification Database; TCGA—The Cancer Genome Atlas; UALCAN—University of ALabama at Birmingham CANcer data analysis Portal.

**Table 2:** Examples of in silico studies of m^1^A and other RNA modification-regulated genes in different types of cancer using the TCGA datasets and other public omics databases and tools.

Study	Cancer Type	Used Datasets	Main Findings
Li et al. [73]	Breast carcinoma (BRCA)	TCGA-BRCA, GSE20685	Eighty-five differentially expressed m^1^A-related genes were observed; six among them were selected as prognostic biomarkers; *MEOX1*, *COL17A1*, *FREM1*, *TNN*, and *SLIT3* were significantly up-regulated in BRCA compared to normal tissues.
Xiao et al. [74]	Liver hepatocellular carcinoma (HCC)	TCGA-LIHC, ICGC-HCC	Two m^6^A/m^5^C/m^1^A-related genes subtypes were identified; a higher tumor mutation burden (TMB) was observed in the high-risk group; high-risk group and patients with higher TMB showed a worse prognosis.
Wu, Shi [75]	Osteosarcoma	TARGET	Risk signature based on m^1^A/m^5^C/m^6^A-associated long non-coding RNAs (lncRNAs) showed a correlation with immune infiltration, cancer microenvironment, and immune-associated genes.
Li et al. [76]	Renal clear cell carcinoma (ccRCC)	TCGA-KIRC, ArrayExpress	Ten m^1^A-regulating genes included in analysis; *YTHDF1*, *TRMT61B*, *TRMT10C*, and *ALKBH1* were identified as prognostic factors; high-risk group has worse survival; checkpoint inhibitors and small drugs A.443654, A.770041, ABT.888, AG.014699, and AMG.706 potentially useful for the high-risk group.
Mao et al. [77]	Glioma	TCGA-GBM, CGGA	Four m^1^A modification-related patterns identified, with clear differences in survival, stemness, genomic heterogeneity, tumor microenvironment (TME), and immune cell infiltration; *PLEK2* and *ABCC3* were screened as the risk-hub genes; *ABCC3* knockdown decreased glioma proliferation and reduced temozolomide (TMZ) resistance.
Wu et al. [78]	Oral squamous cell carcinoma (OSCC)	TCGA-HNSC	Analyzed m^6^A/m^1^A/m^5^C/m^7^G/m^6^Am/Ψ-related genes; found 22 gene signatures; patients divided into low- and high-risk groups, with difference in immune cell infiltration, genetic mutation, and survival potential.

CGGA—Chinese Glioma Genome Atlas; GBM—glioblastoma multiforme; HNSC—head and neck squamous cell carcinoma; ICGC—International Cancer Genome Consortium; KIRC—kidney renal clear cell carcinoma; LIHC—liver hepatocellular carcinoma; m^1^A—N^1^-methyladenosine; m^5^C—5-methylcytosine; m^6^A—N^6^-methyladenosine; m^6^Am—N^6^,2′-O-dimethyladenosine; m^7^G—N^7^-methylguanosine; Ψ—pseudouridine; TARGET—Therapeutically Applicable Research to Generate Effective Treatments; TCGA—The Cancer Genome Atlas.

**Table 3:** Most relevant m^1^A RNA methylation inhibitors and phases of their testing in different cancer types.

Type of Inhibitor	Target Enzyme	Drug Name	Phase of Clinical Trial	Cancer Type	Reference
DNA methyltransferase inhibitors (writer inhibitors)	DNA methyltransferase	azacitidine	FDA-approved	myelodysplastic syndromes, AML	[82]
	DNA methyltransferase	decitabine	FDA-approved	myelodysplastic syndromes, AML	[83]
	METTL3	UZH1a	preclinical	AML, osteosarcoma, kidney	[89]
	METTL3	STM2457	preclinical	AML, neuroblastoma	[90]
	METTL3	STC-15	phase 1	AML	[91]
tRNA methyltransferase inhibitors (writer inhibitor)	TRMT6/TRMT61A	thiram	preclinical	hepatocellular, glioma	[43,108]
RNA demethylase inhibitors (eraser inhibitors)	FTO	rhein	preclinical	AML	[94]
	FTO	MO-I-500	preclinical	breast	[96]
	FTO	FB23-2	preclinical	AML	[97]
	FTO	R-2HG	preclinical	leukemia, glioma	[98]
	FTO	CS1	preclinical	AML	[99]
	FTO	CS2	preclinical	AML	[99]
	FTO	FTO-04	preclinical	glioblastoma	[100]
	FTO	Dac51	preclinical	melanoma	[101]
	ALKBH3	HUHS015	preclinical	prostate	[103]
reader inhibitors	YTHDF YTHDF1	ebselen tegaserod	preclinical preclinical	prostate AML	[104,105]

AML—acute myeloid leukemia; FDA—United States Food and Drug Administration; R-2HG—R-2-hydroxyglutarate.

## Data Availability

No new data were created or analyzed in this study. Data sharing is not applicable to this article.
